# P-1192. Fosmanogepix Expanded Access Experience in Patients With Invasive Aspergillosis

**DOI:** 10.1093/ofid/ofaf695.1385

**Published:** 2026-01-11

**Authors:** Jana Dickter, Alfredo Puing, Ana Fernández Cruz, Jannik Stemler, Apurva Amit Akkad, Juergen Prattes, Ana Belkin, Julie M Steinbrink, Omer E Beaird, Haran Schlamm, Sanjeet S Dadwal, Luis Ostrosky-Zeichner

**Affiliations:** City of Hope National Medical Center, Duarte, California; Yale School of Medicine, New Haven, Connecticut; Hospital Universitario Puerta de Hierro-Majadahonda, Madrid, Madrid, Spain; University of Cologne, Faculty of Medicine and University Hospital Cologne, Department I for Internal Medicine, Excellence Center for Medical Mycology (ECMM), Cologne, NRW, Germany; University of Cologne, Faculty of Medicine and University Hospital Cologne, Institute of Translational Research, Cologne Excellence Cluster on Cellular Stress Responses in Aging-Associated Diseases (CECAD), Cologne, NRW, Germany, Cologne, Nordrhein-Westfalen, Germany; Keck School of Medicine of the University of Southern California, Los Angeles, California; Medical University of Graz, Graz, Steiermark, Austria; Sheba Medical Center, Ramat-Gan, Tel Aviv, Israel; Duke University Medical Center, Durham, NC; University of California Los Angeles, Los Angeles, California; HTS Pharma, San Diego, California; City of Hope National Medical Center, Duarte, California; University of Texas Health Science Center, Houston, Texas

## Abstract

**Background:**

Fosmanogepix (FMGX; prodrug, active moiety manogepix) is the first member of the “gepix” antifungal class. FMGX inhibits Gwt1, depleting GPI-anchor proteins important for fungal cell wall integrity. FMGX shows consistent in vitro activity against *Aspergillus* spp, including azole-resistant strains; it has linear pharmacokinetics with wide tissue distribution, including the central nervous system (CNS). FMGX is available via expanded access (EA) for serious fungal infections in patients with no alternative treatment options (NCT06433128).

**Table 1.**
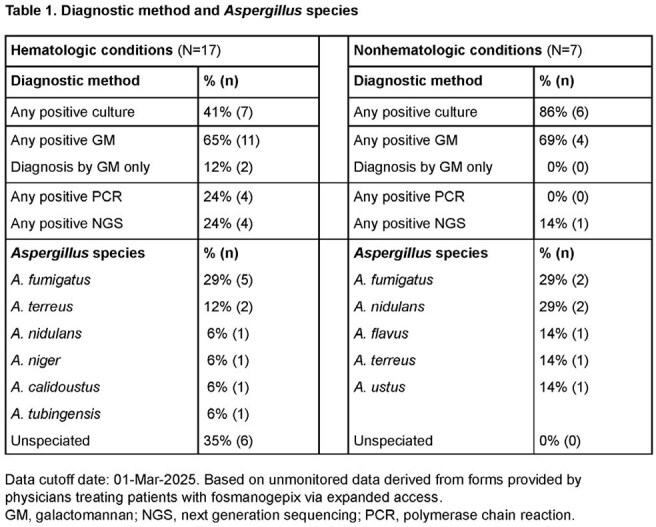
Diagnostic method and Aspergillus species Data cutoff date: 01-Mar-2025. Based on unmonitored data derived from forms provided by physicians treating patients with fosmanogepix via expanded access. GM, galactomannan; NGS, next generation sequencing; PCR, polymerase chain reaction.

**Table 2.**
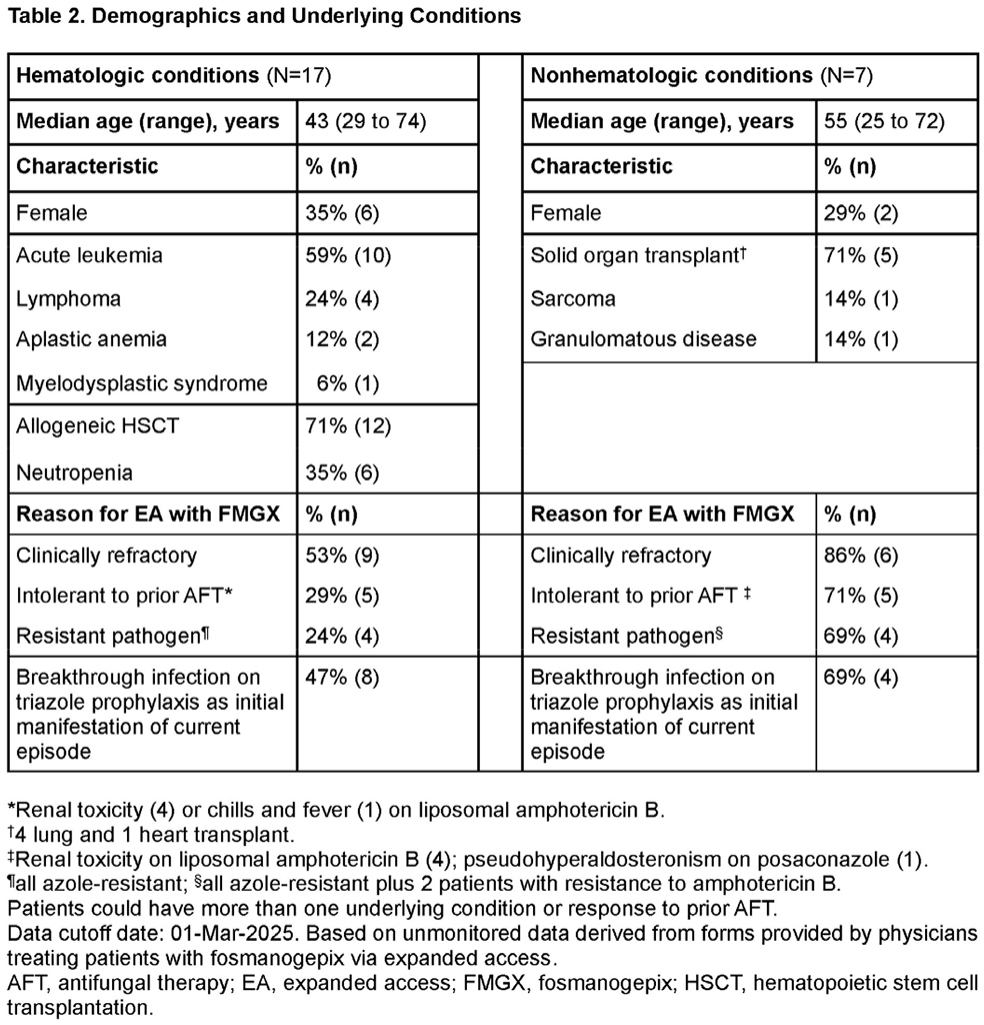
Demographics and Underlying Conditions *Renal toxicity (4) or chills and fever (1) on liposomal amphotericin B. †4 lung and 1 heart transplant. ‡Renal toxicity on liposomal amphotericin B (4); pseudohyperaldosteronism on posaconazole (1). ¶all azole-resistant; §all azole-resistant plus 2 patients with resistance to amphotericin B. Patients could have more than one underlying condition or response to prior AFT. Data cutoff date: 01-Mar-2025. Based on unmonitored data derived from forms provided by physicians treating patients with fosmanogepix via expanded access. AFT, antifungal therapy; EA, expanded access; FMGX, fosmanogepix; HSCT, hematopoietic stem cell transplantation.

**Methods:**

Patients with documented invasive aspergillosis (IA) were treated with FMGX via EA. Data including global response (clinical, radiological, mycological) were collected using structured forms.

**Figure 1.**
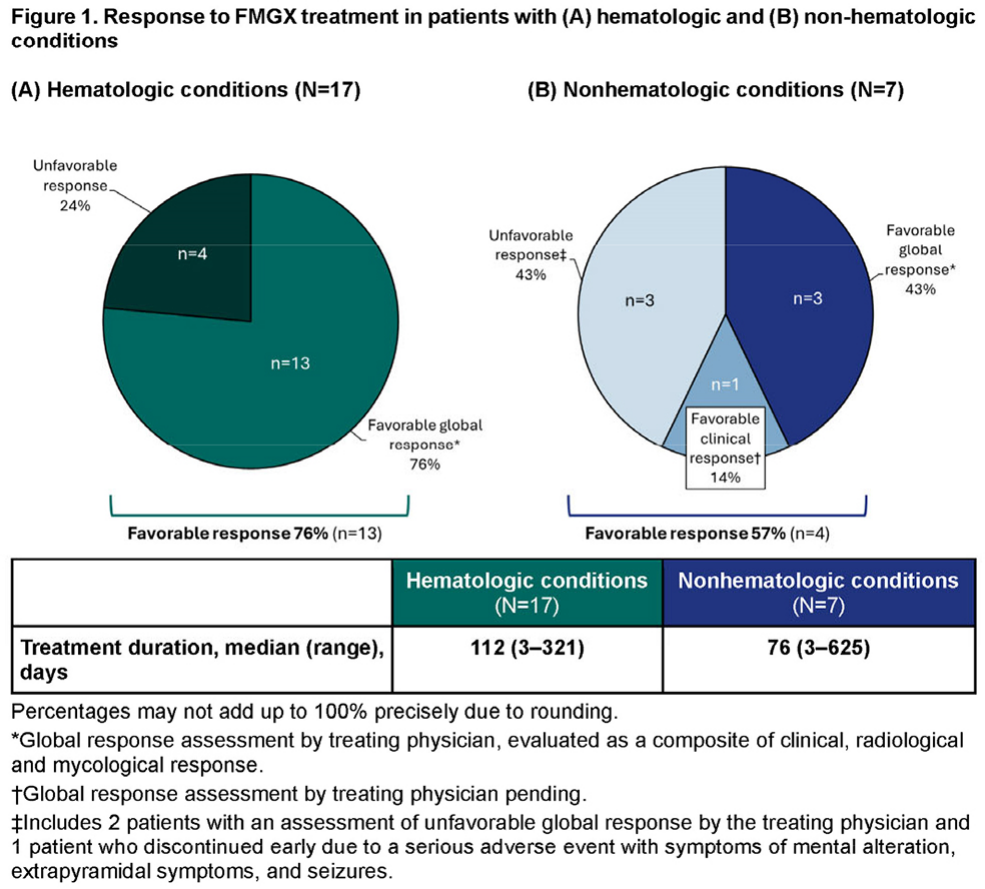
Response to FMGX treatment in patients with (A) hematologic and (B) non-hematologic conditions Percentages may not add up to 100% precisely due to rounding. *Global response assessment by treating physician, evaluated as a composite of clinical, radiological and mycological response. †Global response assessment by treating physician pending. ‡Includes 2 patients with an assessment of unfavorable global response by the treating physician and 1 patient who discontinued early due to a serious adverse event with symptoms of mental alteration, extrapyramidal symptoms, and seizures.

**Results:**

Twenty-four patients (8 females; median age, 45.5 [range, 25–75] y) from 7 countries (63% US) completed FMGX treatment. The most common species was *A. fumigatus* (7), but more patients had non-fumigatus IA (3 *A. nidulans*, 3 *A. terreus*, 3 cryptic *Aspergillus* spp; Table 1).

17 patients had hematologic conditions, mainly acute leukemia (Table 2); 47% had breakthrough IA with posaconazole (5) or isavuconazole (3) prophylaxis. IA was mainly pulmonary (16, including 4 disseminated). Patients were refractory (9) or intolerant (5) to liposomal amphotericin B or had resistant pathogens (4). Response was favorable in 13 patients (76%; Fig 1); 4 patients (24%) died ≤ 6 weeks after starting FMGX. FMGX was well tolerated up to 321 (median 112) days.

7 patients had nonhematologic conditions (Table 2), mostly solid organ transplant (SOT; 5). IA was mainly pulmonary (6, including 1 disseminated). Response was favorable in 4 patients (57%; Fig 1); 3 patients (43%, 2 lung, 1 heart transplant) died ≤ 6 weeks after starting FMGX. FMGX was generally well tolerated up to 625 (median 76) days. One patient discontinued early due to CNS adverse events.

**Conclusion:**

FMGX treatment led to favorable responses in > 70% of patients with mostly pulmonary IA, underlying hematologic malignancies or SOT, breakthrough infections, and infections caused by azole-resistant or non-fumigatus *Aspergillus* spp. FMGX was generally well tolerated for long treatment durations and has potential to be a therapeutic option for IA.

**Disclosures:**

Ana Fernández Cruz, MD, PhD, Astellas: Honoraria|Gilead: Grant/Research Support|Gilead: Honoraria|MSD: Honoraria|Mundipharma: Honoraria|Pfizer: Honoraria Jannik Stemler, MD, AbbVie: Honoraria|Akademie für Infektionsmedizin: Honoraria|Alvea Vax: Advisor/Consultant|Basilea: Grant/Research Support|German Federal Ministry of Education and Research (BMBF): Grant/Research Support|German Society for Infectious Diseases: Travel grants|Gilead: Advisor/Consultant|Gilead: Honoraria|Hikma: Honoraria|Lilly: Honoraria|Meta-Alexander Foundation: Travel grants|Micron Research: Advisor/Consultant|Mundipharma: Honoraria|Noscendo: Grant/Research Support|Pfizer: Honoraria|Scynexis: Grant/Research Support|The Medical Faculty of the University of Cologne: Grant/Research Support Apurva Amit Akkad, MD, Shionogi: Advisor/Consultant Juergen Prattes, MD, AbbVie Inc: Stocks/Bonds (Public Company)|Associates of Cape Cod, Inc.: Honoraria|Gilead: Honoraria|Novo Nordisk: Stocks/Bonds (Public Company)|Pfizer: Honoraria|Sobi: Honoraria Julie M. Steinbrink, MD, MHS, Biomeme: patents for gene expression classifiers of fungal infection|McGraw Hill Publishing: royalties Haran Schlamm, MD, Amplyx: Advisor/Consultant|Basilea: Advisor/Consultant|Pfizer: Advisor/Consultant Sanjeet S. Dadwal, MD, Ansun Biopharma: Grant/Research Support|Aseptiscope, Inc.: Stocks/Bonds (Private Company)|Basilea: Advisor/Consultant|Basilea: Grant/Research Support|F2G: Grant/Research Support|Karius: Advisor/Consultant|Karius: Grant/Research Support|Karius: Honoraria|Merck: Advisor/Consultant|Pfizer: Grant/Research Support|Pulmotect: Grant/Research Support|Symbio: Grant/Research Support|Takeda: Advisor/Consultant Luis Ostrosky-Zeichner, MD, Basilea: Advisor/Consultant|Basilea: Grant/Research Support|Basilea: Honoraria|Eurofins Viracor: Advisor/Consultant|Eurofins Viracor: Grant/Research Support|Eurofins Viracor: Honoraria|F2G: Advisor/Consultant|F2G: Grant/Research Support|F2G: Honoraria|Gilead: Advisor/Consultant|Gilead: Grant/Research Support|Gilead: Honoraria|GSK: Advisor/Consultant|GSK: Grant/Research Support|GSK: Honoraria|Melinta: Advisor/Consultant|Melinta: Grant/Research Support|Melinta: Honoraria|Pfizer: Advisor/Consultant|Pfizer: Grant/Research Support|Pfizer: Honoraria|Pulmocide: Advisor/Consultant|Pulmocide: Grant/Research Support|Pulmocide: Honoraria|Scynexis: Advisor/Consultant|Scynexis: Grant/Research Support|Scynexis: Honoraria|T2 Biosystems: Advisor/Consultant|T2 Biosystems: Grant/Research Support|T2 Biosystems: Honoraria

